# Impacto do Envelhecimento Populacional na Prevalência de Doenças Cardiovasculares nas Macrorregiões do Brasil: Previsões para 2050

**DOI:** 10.36660/abc.20250699

**Published:** 2026-05-21

**Authors:** Rafael Rauber, Aline Mânica, Clodoaldo Antônio de Sá, Fátima Kremer Ferretti, Junir Antônio Lutinski, Maria Assunta Busato, Samuel Spiegelberg Zuge, Sinval Adalberto Rodrigues, Vanessa da Silva Corralo, Thiago André Carniel

**Affiliations:** 1 Universidade Comunitária da Região de Chapecó Chapecó SC Brasil Universidade Comunitária da Região de Chapecó - Programa de Pós-Graduação em Ciências da Saúde, Chapecó, SC – Brasil

**Keywords:** Doenças Cardiovasculares, Prevalência, Modelos Epidemiológicos

## Abstract

As doenças cardiovasculares (DCV) têm sido consistentemente a principal causa de morbidade e mortalidade em todo o mundo, com um aumento significativo impulsionado pelo envelhecimento demográfico. Embora o Brasil reflita esse padrão global, esse ônus pode estar distribuído de forma desigual entre suas macrorregiões. O principal objetivo deste trabalho é investigar as tendências de prevalência de DCV nas macrorregiões do Brasil de 1990 a 2021 e prever cenários até 2050, considerando o envelhecimento demográfico. As taxas de prevalência bruta e padronizada por idade foram obtidas do estudo *Global Burden of Disease*. Modelos neurais de espaço de estados foram utilizados para prever as tendências até 2050, com intervalos de confiança de 95% estimados por meio de uma abordagem semelhante à de Monte Carlo. As taxas de prevalência bruta tendem a aumentar substancialmente em todas as macrorregiões até 2050, com a região Centro-Oeste apresentando o maior aumento (64,6% [52,3-79,5]) e a região Nordeste o menor (39,2% [33,4-46,5]). Em contrapartida, se espera que as taxas padronizadas por idade permaneçam praticamente constantes ao longo dos anos, com apenas um modesto aumento observado no Nordeste (<7%). Mesmo com taxas padronizadas por idade relativamente constantes, se prevê que os sistemas de saúde enfrentem crescentes desafios clínicos e econômicos associados a uma população mais idosa e com maior número de comorbidades. As disparidades regionais reforçam a necessidade de estratégias de prevenção específicas para mitigar o impacto das DCV no Brasil.

## Introdução

As doenças cardiovasculares (DCV) continuam sendo a principal causa de incapacidade e mortalidade em todo o mundo, sendo responsáveis por aproximadamente 20,5 milhões de mortes em 2021, ou seja, um terço da mortalidade global total.^1,2^ O Brasil reflete essa tendência global, com as DCVs sendo a principal causa de morte até 2019.^3^ Embora a COVID-19 tenha superado temporariamente esse número em 2021, cerca de 6,9% da população brasileira ainda convivia com DCVs naquele mesmo ano.^4^

Esses números destacam um fardo social e econômico significativo, em grande parte atribuível ao rápido envelhecimento da população.^5^ Até 2050, se espera que o número de pessoas com 60 anos ou mais dobre, e o número de pessoas com mais de 80 anos triplique.^6^ Consequentemente, se prevê um aumento substancial no número absoluto de casos de DCVs, impulsionado principalmente por essa mudança demográfica em direção a uma população em envelhecimento.^7^

Embora ações imediatas sejam essenciais para combater as DCV, estimativas futuras são necessárias para apoiar profissionais de saúde e formuladores de políticas na tomada de decisões para intervenções oportunas. Motivados por isso, examinamos a prevalência de DCV nas macrorregiões do Brasil de 1990 a 2021 e projetamos cenários até 2050, considerando o envelhecimento demográfico.

## Métodos

### Conjunto de dados

As pirâmides etárias foram construídas com base em dados obtidos das estimativas populacionais das Nações Unidas.^8^ Os dados sobre DCVs foram obtidos do *Global Burden of Disease* (GBD), um estudo internacional que conta com importantes colaborações no Brasil.^4,9^ Especificamente, utilizamos as taxas de prevalência de DCV em todas as idades (valores brutos) e as taxas de prevalência padronizadas por idade de 1990 a 2021 nas cinco macrorregiões do Brasil. Os dados abrangem indivíduos de ambos os sexos.

### Desenvolvimento dos modelos e estatísticas

Utilizamos modelos neurais de espaço de estados para prever tendências até 2050. Esses modelos são um tipo robusto de modelos não lineares que integram a teoria clássica de espaço de estados com redes neurais (aprendizado profundo).^10^ As incertezas das previsões foram estimadas por meio de um método semelhante ao de Monte Carlo. Os resultados são apresentados como medianas com intervalos de confiança de 95%, e todos os cálculos foram realizados utilizando o Matlab (R2024b, MathWorks, Inc.).

Embora os dados de 2020-2021 possam estar potencialmente enviesados devido à pandemia de COVID-19, verificamos que a inclusão ou exclusão desse pequeno número de observações não introduz desvios estruturais substanciais capazes de alterar significativamente as previsões de longo prazo.

## Resultados

**Figura 1:**
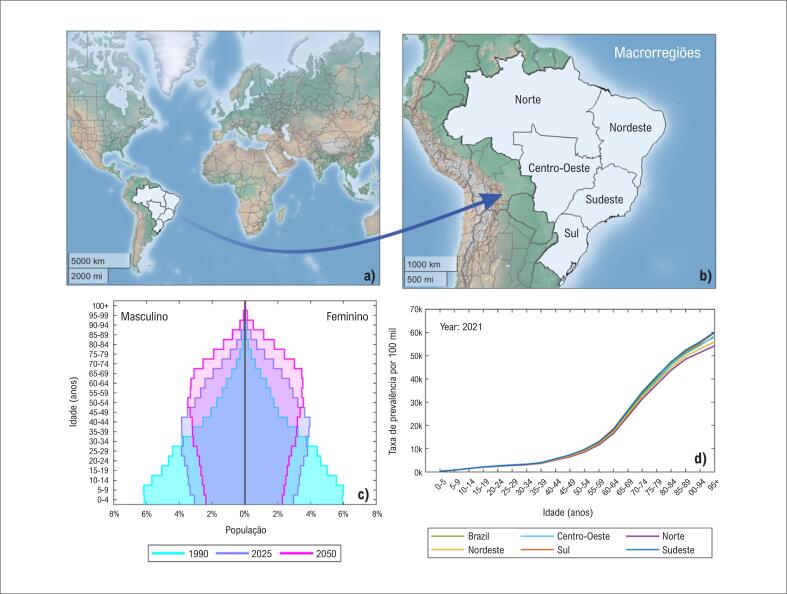
a) Localização geográfica global do Brasil. b) Divisão geográfica das cinco macrorregiões brasileiras. c) Pirâmides etárias do Brasil em 1990, com estimativas para 2025 e 2050, destacam as mudanças demográficas em direção ao envelhecimento populacional. Até 2050, espera-se que uma parcela substancial da população brasileira esteja na faixa etária de meia-idade e idosos. d) Taxas de prevalência de DCVs em diferentes faixas etárias para as cinco macrorregiões do Brasil em 2021.

As pirâmides etárias do Brasil mostram uma clara tendência de envelhecimento populacional (Figura 1c). As taxas de prevalência de DCVs em 2021 apresentam padrões consistentes em todas as regiões, com um aumento significativo e progressivo entre indivíduos com 60 anos ou mais (Figura 1d). Por exemplo, as taxas de prevalência entre indivíduos de 60 a 64 anos e de 80 a 84 anos são aproximadamente 6 e 16 vezes maiores, respectivamente, do que as de indivíduos de 25 a 29 anos.

**Figura 2:**
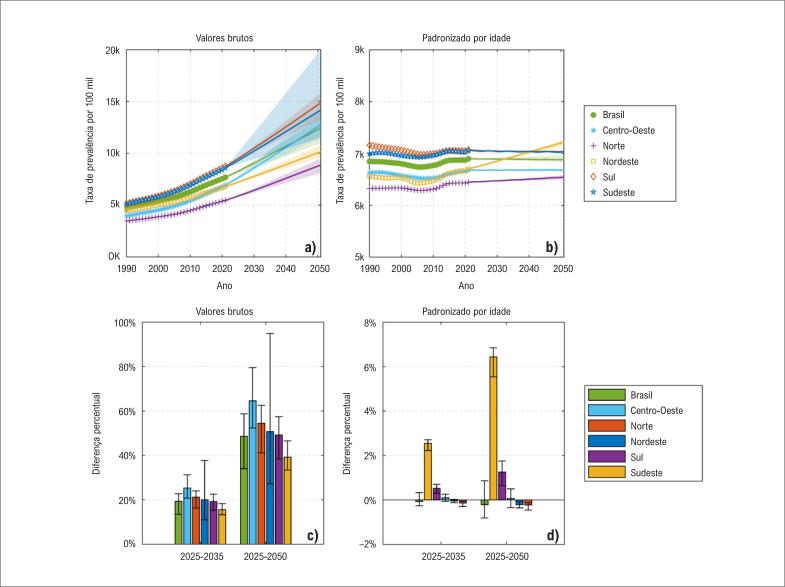
a-b) Taxas de prevalência (todas as idades e padronizadas por idade) e respectivas previsões para o Brasil e suas cinco macrorregiões. c-d) Aumento ou diminuição percentual para uma previsão de 10 anos (2025-2035) e estimativas de 25 anos (2025-2050), calculadas a partir das curvas previstas.

As taxas de prevalência brutas e padronizadas por idade de 1990 a 2021, com previsões até 2050, são apresentadas nas Figuras 2a e 2b. Espera-se que as taxas brutas aumentem consistentemente até 2050. Em contraste, as taxas padronizadas por idade mantêm padrões relativamente constantes, com exceção da região nordeste, que apresenta uma leve tendência de alta até 2050.

Para uma previsão de 10 anos (Figura 2c), a taxa de prevalência geral de DCV no Brasil deverá aumentar em 19,3% [13,5-22,8]. A região Centro-Oeste deverá apresentar o maior aumento, de 25,3% [20,7-31,2], enquanto a região Nordeste deverá ter o menor aumento, de 15,6% [13,3-18,3]. As regiões Sul, Sudeste e Norte deverão apresentar aumentos de 21,2% [16,3-24,0], 20,0% [11,0-37,8] e 19,2% [15,3-22,6], respectivamente.

Olhando para os próximos 25 anos, se prevê que a taxa de prevalência no Brasil aumente em 48,6% [34,0-58,7]. As tendências nas macrorregiões refletem as da previsão de 10 anos, com a região Centro-Oeste apresentando novamente o maior aumento previsto, de 64,6% [52,3-79,5], e a região Nordeste o menor, de 39,2% [33,4-46,5]. Espera-se que as regiões Sul, Sudeste e Norte aumentem em 54,5% [41,1-62,5], 50,7% [27,2-94,9] e 49,2% [38,4-57,4], respectivamente.

Com relação às taxas padronizadas por idade (Figura 2d), espera-se um aumento percentual modesto para o nordeste até 2050 (< 7%), e as demais regiões apresentam variações muito pequenas (< 2%).

## Discussão

Os resultados indicam um aumento significativo na taxa de prevalência geral de DCV em todas as macrorregiões brasileiras até 2050, enquanto as taxas padronizadas por idade devem permanecer estáveis, com um aumento modesto previsto apenas para o Nordeste. Esse aparente paradoxo surge da mudança demográfica em direção a uma população mais idosa. À medida que mais indivíduos atingem idades avançadas, associadas a um maior risco de DCV, a carga total da doença aumenta, mesmo quando os fatores de risco em nível populacional são controlados de forma eficaz.^2,3,6^

A estabilidade das taxas padronizadas por idade ressalta o papel central da mudança demográfica na definição da prevalência futura. Conforme verificado em 2021, a taxa de prevalência entre indivíduos de 80 a 84 anos foi quase 16 vezes maior do que entre aqueles de 25 a 29 anos, demonstrando o acentuado gradiente etário que impulsiona o aumento futuro nos números absolutos. Outros estudos confirmaram esse padrão, mostrando que o envelhecimento populacional resulta em altas taxas de DCVs em faixas etárias mais avançadas, acompanhadas de multimorbidade, fragilidade e necessidades terapêuticas complexas.^7,11,12^ Mesmo que as taxas padronizadas por idade permaneçam constantes ou diminuam ligeiramente, o envelhecimento da população por si só garante que o número absoluto de casos continuará a crescer.

A heterogeneidade regional também contribui para o ônus nacional. Embora tenham ocorrido melhorias na prevenção e no tratamento em todo o país, elas não foram distribuídas de forma uniforme (Figura 2b). Pesquisas populacionais confirmam diferenças persistentes nos indicadores de saúde cardiovascular, com menor alcance das metas ideais em regiões com condições socioeconômicas menos favoráveis.^9,13,14^ Esses achados são consistentes com o aumento persistente na prevalência de DCV padronizada por idade observado no Nordeste (Figura 2b), a região brasileira com os indicadores socioeconômicos mais desfavoráveis em relação às demais. Portanto, essas desigualdades sugerem que o efeito demográfico do envelhecimento interagirá com as disparidades regionais, amplificando a futura carga da doença.

O aumento previsto na prevalência acarreta importantes implicações econômicas e sociais. Análises da Europa mostram que o custo relacionado às DCVs já representa 2% do produto interno bruto (PIB), impulsionado principalmente pelas demandas de uma população em envelhecimento.^15^ No Brasil, estudos anteriores estimaram os custos relacionados às DCVs em cerca de 0,7% do PIB, com riscos alimentares e controle inadequado de fatores modificáveis contribuindo substancialmente para esse percentual.^16–18^ Com o envelhecimento contínuo da população, os sistemas de saúde enfrentarão não apenas um número crescente de pacientes, mas também a complexidade dos cuidados necessários para a população idosa com múltiplas comorbidades.

Apesar da consistência dos nossos resultados com os dados internacionais e nacionais reportados, este estudo apresenta limitações. As previsões foram realizadas exclusivamente com base nos dados do GBD; portanto, dependem das premissas demográficas, que podem influenciar as estimativas futuras de prevalência. Além disso, a persistência das disparidades regionais no controle dos fatores de risco e no acesso aos cuidados de saúde pode interagir com as tendências demográficas de maneiras não totalmente capturadas pelo modelo. Mais importante ainda, o conjunto de dados subjacente pode mudar a cada atualização do banco de dados do GBD, uma vez que as estimativas revisadas incorporam novas fontes de dados e refinamentos metodológicos. Portanto, estudos como este devem ser revisitados periodicamente à medida que novas atualizações do GBD se tornam disponíveis. Estudos futuros devem explorar padrões específicos por sexo, indicadores epidemiológicos adicionais e abordagens de modelagem multivariada mais robustas que incorporem explicitamente os principais fatores de risco cardiovascular, permitindo uma compreensão mais profunda de como tendências temporais opostas podem moldar conjuntamente a futura carga da doença.

## Data Availability

Os conteúdos estão disponíveis no link http://ghdx.healthdata.org/
