# Supplementary material for: Impacto do Envelhecimento Populacional na Prevalência de Doenças Cardiovasculares nas Macrorregiões do Brasil: Previsões para 2050
Source: Arq Bras Cardiol. 2026 May 21;123(4):e20250699. [Article in Portuguese] doi: 10.36660/abc.20250699 (PMC13398802; doi:10.36660/abc.20250699)
Supplement: Translation

# Translation: Impact of Population Aging on Cardiovascular Disease Prevalence in the Macroregions of Brazil: Forecasts to 2050

**DOI:** 10.36660/abc.20250699i

**Authors:** Rafael Rauber, Aline Mânica, Clodoaldo Antônio de Sá, Fátima Kremer Ferretti, Junir Antônio Lutinski, Maria Assunta Busato, Samuel Spiegelberg Zuge, Sinval Adalberto Rodrigues, Vanessa da Silva Corralo, Thiago André Carniel

**Affiliations:** 1 Chapecó SC Brazil Universidade Comunitária da Região de Chapecó - Programa de Pós-Graduação em Ciências da Saúde, Chapecó, SC – Brazil

**Keywords:** Cardiovascular Diseases, Prevalence, Epidemiological Models

## Abstract

Cardiovascular diseases (CVD) have consistently been the leading cause of morbidity and mortality worldwide, with an increasing burden driven by demographic aging. Although Brazil mirrors this global pattern, this burden may be unevenly distributed across its macroregions. The main objective of this work is to investigate the trends in CVD prevalence in the macroregions of Brazil from 1990 to 2021 and to forecast scenarios to 2050, considering demographic aging. Crude and age-standardized prevalence rates were obtained from the Global Burden of Disease study. Neural state-space models were used to forecast trends to 2050, with 95% confidence intervals estimated via a Monte Carlo-like approach. Crude prevalence rates tend to rise substantially across all macroregions until 2050, with the central-west expected to see the highest rise (64.6% [52.3-79.5]) and the northeast the lowest (39.2% [33.4-46.5]). In contrast, age-standardized rates are expected to remain largely constant across the years, with only a modest increase observed in the northeast (<7%). Even under relatively constant age-standardized rates, health systems are expected to face growing clinical and economic challenges associated with an older and more multimorbid population. Regional disparities underscore the need for tailored prevention strategies to mitigate the burden of CVD in Brazil.

## Introduction

Cardiovascular disease (CVD) has remained the leading cause of disability and mortality worldwide, accounting for approximately 20.5 million deaths in 2021, i.e., one-third of total global mortality.^1,2^ Brazil mirrors this global trend, with CVD being the primary cause of death up to 2019.^3^ Although COVID-19 temporarily surpassed them in 2021, around 6.9% of the Brazilian population still lived with CVD in that same year.^4^

These figures highlight a significant social and economic burden, largely attributable to the rapid aging of the population.^5^ By 2050, the number of individuals aged 60 and above is expected to double, and those over 80 to triple.^6^ Consequently, a substantial increase in the absolute number of CVD cases is anticipated, primarily driven by this demographic shift toward an aging population.^7^

While immediate actions are essential to combat CVD, future estimates are needed to support healthcare professionals and policymakers in decision-making for timely interventions. Motivated by this, we examined the CVD prevalence in the macroregions of Brazil from 1990 to 2021, and forecasted scenarios to 2050, considering demographic aging.

## Methods

### Dataset

Age pyramids were constructed based on data obtained from the United Nations population estimates.^8^ Cardiovascular disease data were obtained from the Global Burden of Disease (GBD), an international study holding important collaborations in Brazil.^4,9^ Specifically, we leveraged CVD prevalence rates at all ages (crude values) and age-standardized prevalence rates from 1990 to 2021 in the five macroregions of Brazil. Data encompasses both male and female individuals.

### Model development and statistics

We employed neural state-space models to forecast trends to 2050. These are a robust type of nonlinear models which integrate classical state-space theory with neural networks (deep learning).^10^ Prediction uncertainties were estimated through a Monte Carlo-like method. Results are reported as medians with 95% confidence intervals, and all computations were conducted using Matlab (R2024b, MathWorks, Inc.).

Although data from 2020-2021 could be potentially biased due to the COVID-19 pandemic, we verified that the inclusion or exclusion of this small number of observations does not introduce substantial structural deviations capable of meaningfully altering long-term forecasts.

## Results

Age pyramids of Brazil show a clear trend towards population aging (Figure 1c). Cardiovascular disease prevalence rates in 2021 present consistent patterns across all regions, with a significant and progressive increase among individuals aged 60 years and older (Figure 1d). For instance, the prevalence rates among individuals aged 60-64 and 80-84 are approximately 6 and 16 times higher than those aged 25-29, respectively.

**Figure 1:** a) Global geographic location of Brazil. b) Geographic division of the five Brazilian macroregions. c) Age pyramids for Brazil in 1990, with estimates for 2025 and 2050, highlight demographic shifts towards population aging. By 2050, a substantial proportion of the Brazilian population is expected to fall within the middle-aged and older adults categories. d) Prevalence rates of cardiovascular disease (CVD) across different age groups for the five macroregions of Brazil in 2021.

The crude and age-standardized prevalence rates from 1990 to 2021, with forecasts extending to 2050, are shown in Figures 2a and 2b. Crude rates are expected to rise consistently until 2050. In contrast, age-standardized rates maintain relatively constant patterns, apart from the northeast, which presents a modest upward trend towards 2050.

**Figure 2:** a-b) Prevalence rates (all ages and age-standardized) and related forecasts for Brazil and its five macroregions. c-d) Percentage increase or decrease for a 10-year forecast (2025-2035) and 25-year estimates (2025-2050), computed from the predicted curves.

For a 10-year forecast (Figure 2c), the overall CVD prevalence rate in Brazil is expected to rise by 19.3% [13.5-22.8]. The central-west region is expected to see the largest increase at 25.3% [20.7-31.2], while the northeast is anticipated to have the smallest rise at 15.6% [13.3-18.3]. The south, southeast, and north regions are expected to increase by 21.2% [16.3-24.0], 20.0% [11.0-37.8], and 19.2% [15.3-22.6], respectively.

Looking ahead 25 years, the prevalence rate in Brazil is predicted to increase by 48.6% [34.0-58.7]. The trends across the macroregions mirror those of the 10-year forecast, with the central-west again showing the highest anticipated increase at 64.6% [52.3-79.5], and the northeast the lowest at 39.2% [33.4-46.5]. The south, southeast, and north regions are expected to increase by 54.5% [41.1-62.5], 50.7% [27.2-94.9], and 49.2% [38.4-57.4], respectively.

Regarding age-standardized rates (Figure 2d), a modest percentage increase is expected for the northeast by 2050 (< 7%), and the remaining regions exhibit very small variations (< 2%).

## Discussion

The results indicate a significant increase in the overall CVD prevalence rate across all Brazilian macroregions by 2050, while age-standardized rates are expected to remain stable, with a modest rise predicted only for the northeast. This apparent paradox arises from the demographic shift towards an older population. As more individuals reach advanced ages, associated with a higher risk of CVD, the total disease burden increases, even as population-level risk factors are managed effectively.^2,3,6^

The stability of age-standardized rates underscores the central role of demographic change in shaping future prevalence. As verified in 2021, the prevalence rate among individuals aged 80–84 years was almost 16 times higher than among those aged 25–29, demonstrating the steep age gradient that drives the future rise in crude numbers. Other studies have confirmed this pattern, showing that aging populations experience high CVD rates in older age groups, accompanied by multimorbidity, frailty, and complex therapeutic needs.^7,11,12^ Even if age-standardized rates remain constant or decline slightly, population aging alone ensures that the absolute number of cases will continue to grow.

Regional heterogeneity also contributes to the national burden. Although improvements in prevention and treatment have occurred throughout the country, they have not been evenly distributed (Figure 2b). Population-based surveys confirm persistent differences in cardiovascular health indicators, with lower attainment of ideal targets in regions with less favorable socioeconomic conditions.^9,13,14^ These findings are consistent with the persistent increase in age-standardized CVD prevalence observed in the Northeast (Figure 2b), the Brazilian region with the most adverse socioeconomic indicators relative to the others. Therefore, these inequalities suggest that the demographic effect of aging will interact with regional disparities, amplifying the future burden of the disease.

The expected increase in prevalence carries important economic and social implications. Analyses from Europe show that the cost related to CVD already accounts for 2% of the gross domestic product (GDP), largely driven by the demands of an aging population.^15^ In Brazil, earlier studies estimated CVD-related costs around 0.7% of GDP, with dietary risks and inadequate control of modifiable factors contributing substantially.^16–18^ As the population continues to age, health systems will face not only a growing number of patients but also the complexity of care required for the multimorbid older population.

Despite the consistency of our findings with international and national reported data, this study has limitations. The forecasts were performed solely using GBD data; therefore, they are contingent on the demographic assumptions, which may influence future prevalence estimates. Moreover, the persistence of regional disparities in risk factors control and healthcare access may interact with demographic trends in ways not fully captured by the model. Most importantly, the underlying dataset may change with each update of the GBD database, as revised estimates incorporate new data sources and methodological refinements. Therefore, studies such as this should be periodically revisited as new GBD updates become available. Future studies should explore sex-specific patterns, additional epidemiological indicators, and more robust multivariate modeling approaches that explicitly incorporate major cardiovascular risk factors, enabling a deeper understanding of how opposing temporal trends may jointly shape future disease burden.

## Data Availability

The content is available at the link http://ghdx.healthdata.org/
